# Supplementary figures and images for: Recovering mixtures of fast-diffusing states from short single-particle trajectories
Source: eLife. 2022 Sep 6;11:e70169. doi: 10.7554/eLife.70169 (PMC9451534; doi:10.7554/eLife.70169)

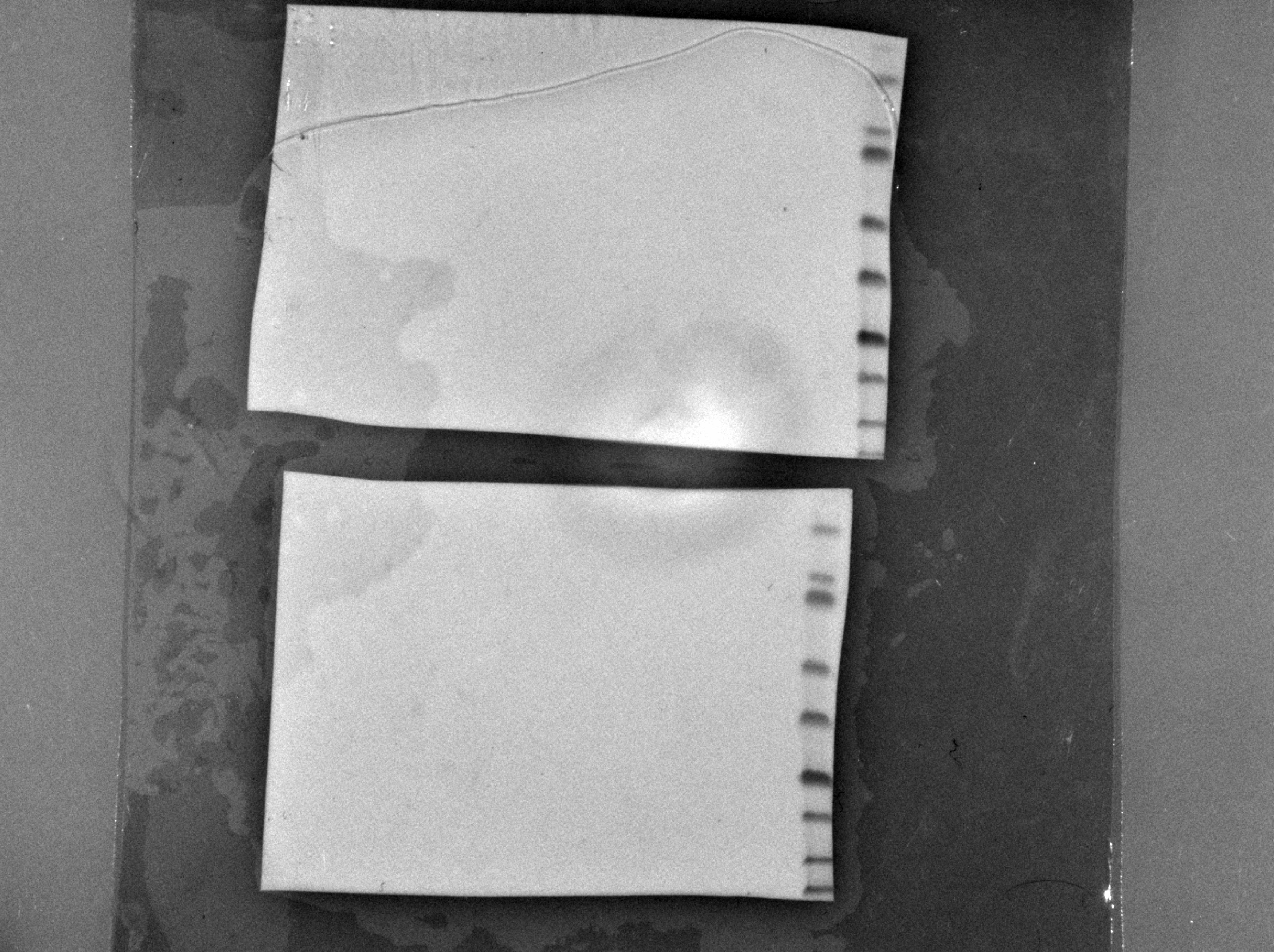

Supplement: Figure 5—source data 1. [file elife-70169-fig5-data1.zip › Figure 5-Source Data 1.png]

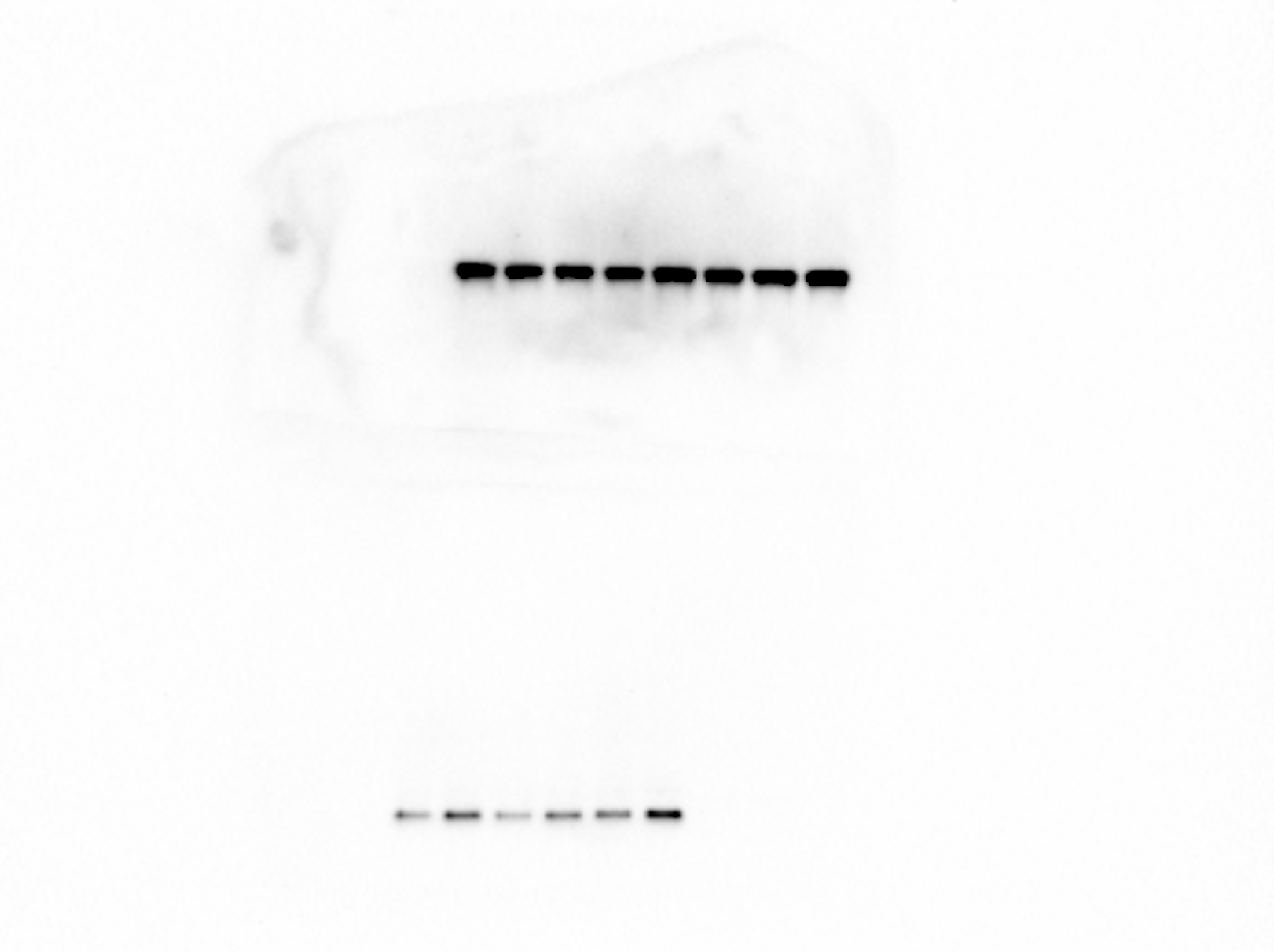

Supplement: Figure 5—source data 1. [file elife-70169-fig5-data1.zip › Figure 5-Source Data 2.png]

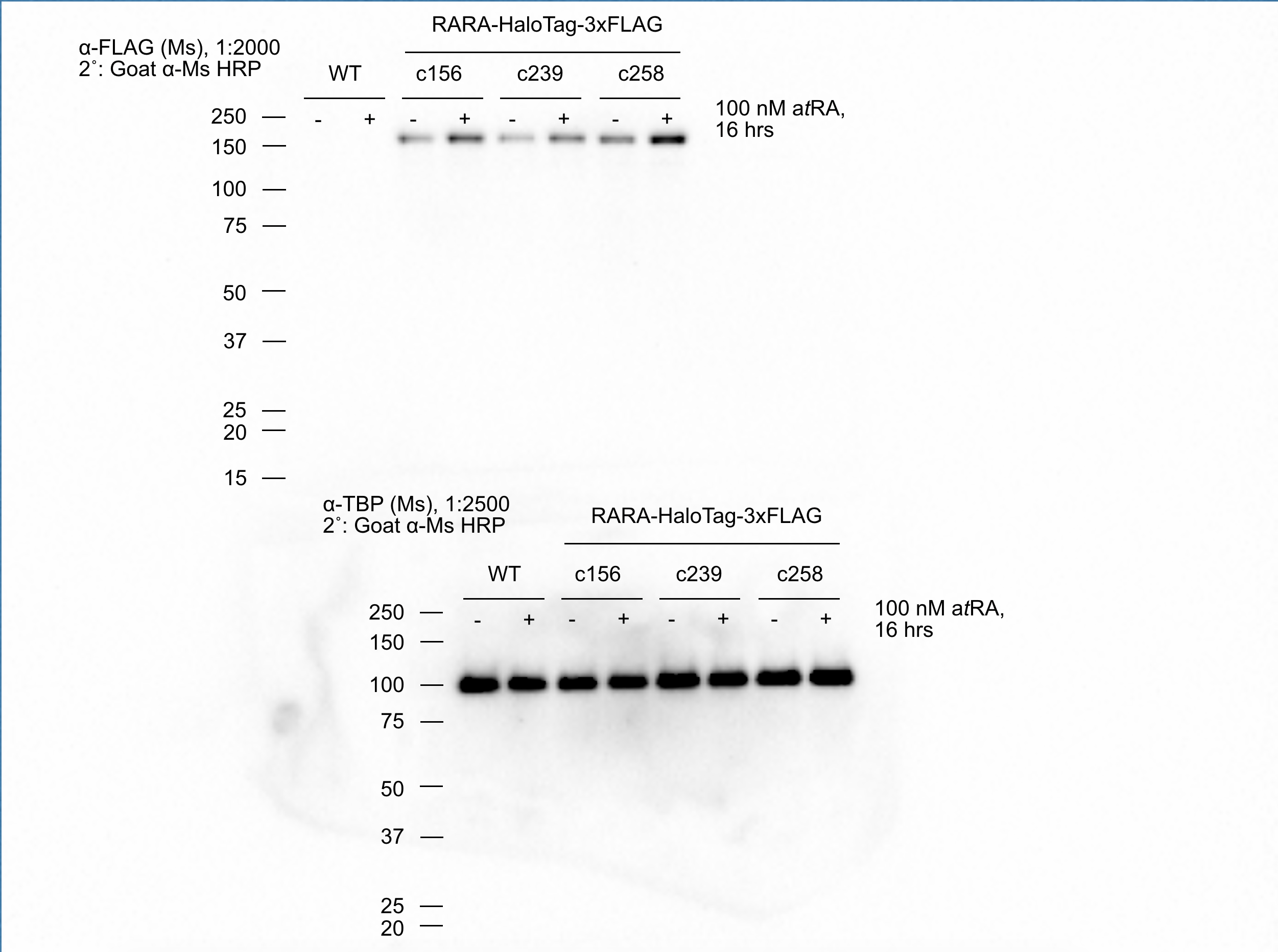

Supplement: Figure 5—source data 1. [file elife-70169-fig5-data1.zip › Figure 5-Source Data 3.png]

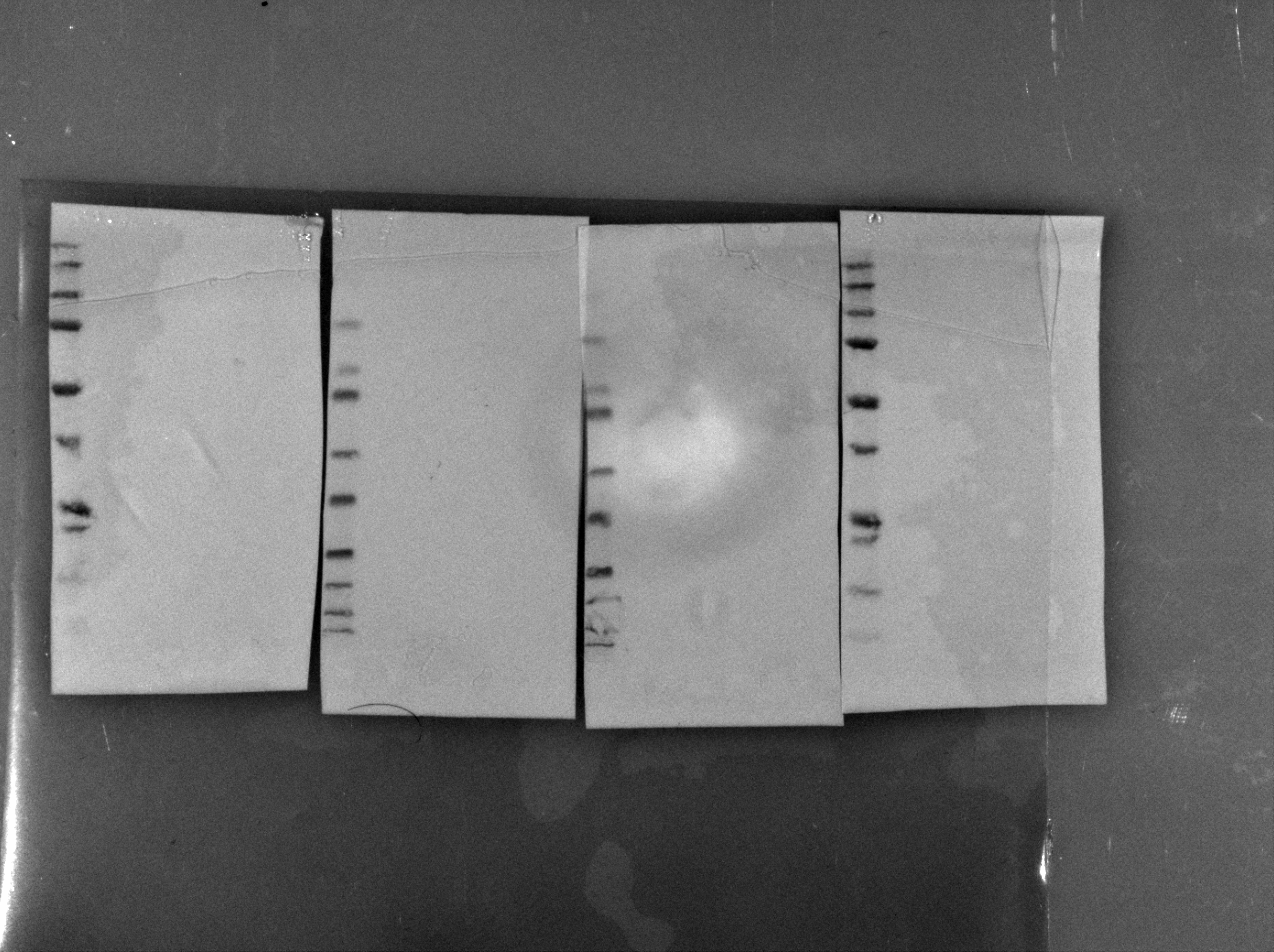

Supplement: Figure 6—figure supplement 1—source data 1. [file elife-70169-fig6-figsupp1-data1.zip › Figure 6-Figure Supplement 1-Source Data 1.tif]

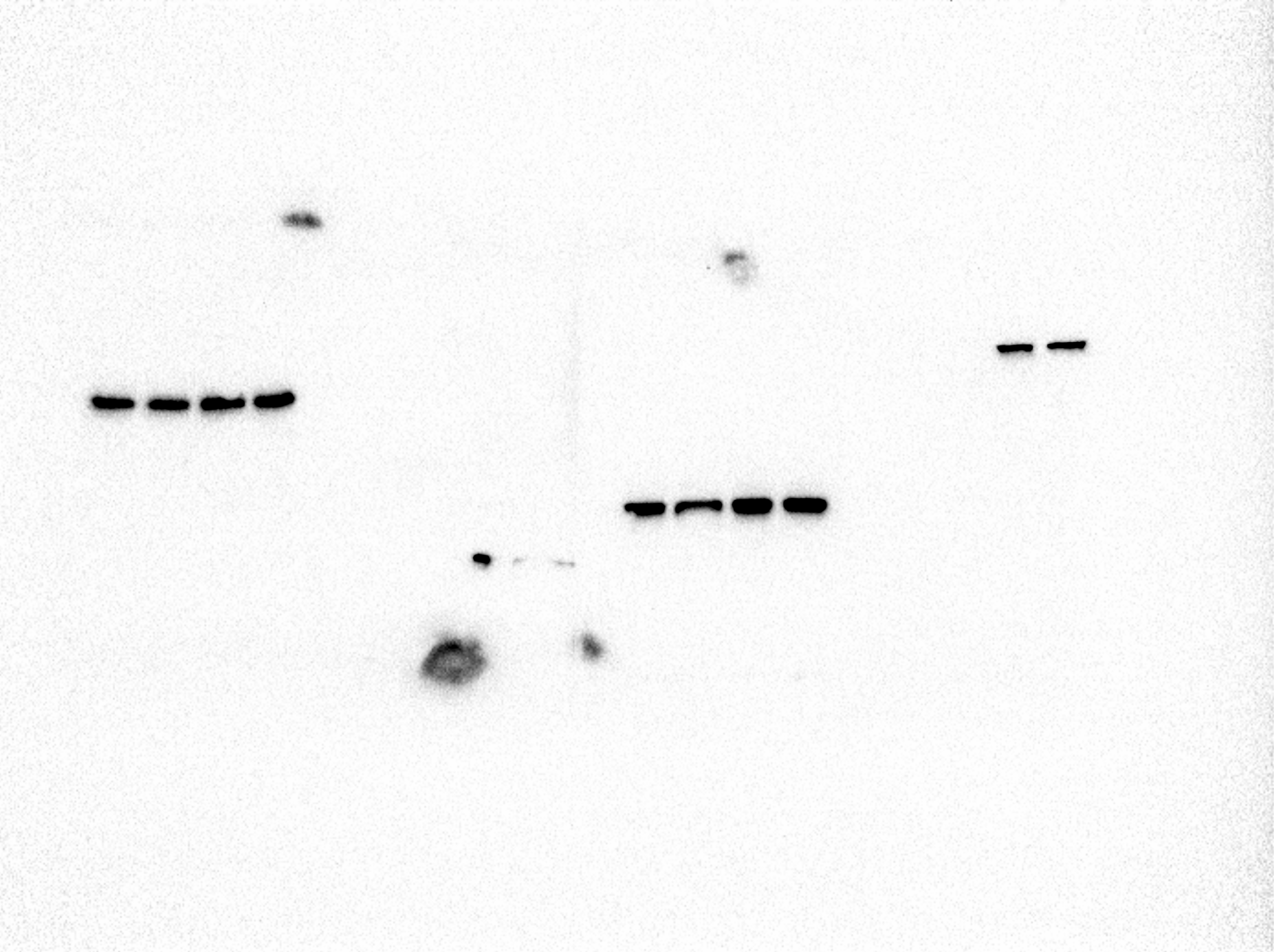

Supplement: Figure 6—figure supplement 1—source data 1. [file elife-70169-fig6-figsupp1-data1.zip › Figure 6-Figure Supplement 1-Source Data 2.tif]

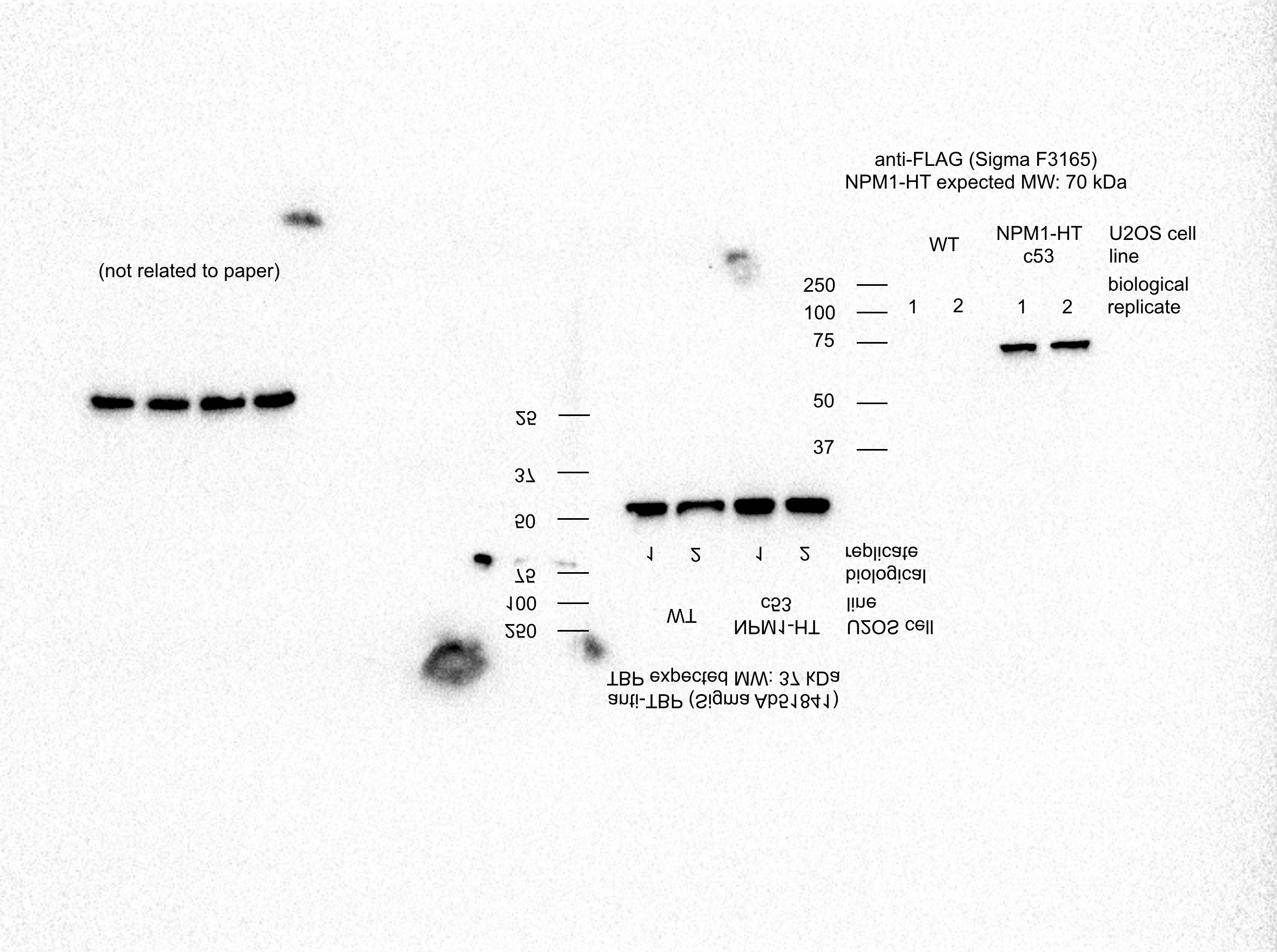

Supplement: Figure 6—figure supplement 1—source data 1. [file elife-70169-fig6-figsupp1-data1.zip › Figure 6-Figure Supplement 1-Source Data 3.png]
