# Supplementary material for: Recovering mixtures of fast-diffusing states from short single-particle trajectories
Source: eLife. 2022 Sep 6;11:e70169. doi: 10.7554/eLife.70169 (PMC9451534; doi:10.7554/eLife.70169)
Supplement: Transparent reporting form [file elife-70169-transrepform1.docx]

***eLife’s* transparent reporting form**

We encourage authors to provide detailed information *within their submission* to facilitate the interpretation and replication of experiments. Authors can upload supporting documentation to indicate the use of appropriate reporting guidelines for health-related research (see [EQUATOR Network](http://www.equator-network.org/%20)), life science research (see the [BioSharing Information Resource](https://biosharing.org/)), or the [ARRIVE guidelines](http://www.plosbiology.org/article/info:doi/10.1371/journal.pbio.1000412) for reporting work involving animal research. Where applicable, authors should refer to any relevant reporting standards documents in this form.

If you have any questions, please consult our Journal Policies and/or contact us: [editorial@elifesciences.org](mailto:editorial@elifesciences.org).

**Sample-size estimation**

- You should state whether an appropriate sample size was computed when the study was being designed
- You should state the statistical method of sample size computation and any required assumptions
- If no explicit power analysis was used, you should describe how you decided what sample (replicate) size (number) to use

Please outline where this information can be found within the submission (e.g., sections or figure legends), or explain why this information doesn’t apply to your submission:

The sample sizes relevant are: the number of biological replicates for each experiment, the number of SPT experiments per biological replicate, and the number of trajectories observed per SPT experiment. All of these are outlined in the relevant figure legends.

**Replicates**

- You should report how often each experiment was performed
- You should include a definition of biological versus technical replication
- The data obtained should be provided and sufficient information should be provided to indicate the number of independent biological and/or technical replicates
- If you encountered any outliers, you should describe how these were handled
- Criteria for exclusion/inclusion of data should be clearly stated
- High-throughput sequence data should be uploaded before submission, with a private link for reviewers provided (these are available from both GEO and ArrayExpress)

Please outline where this information can be found within the submission (e.g., sections or figure legends), or explain why this information doesn’t apply to your submission:

The number of replicates (both technical and biological) are always labelled in figure legends.

**Statistical reporting**

- Statistical analysis methods should be described and justified
- Raw data should be presented in figures whenever informative to do so (typically when N per group is less than 10)
- For each experiment, you should identify the statistical tests used, exact values of N, definitions of center, methods of multiple test correction, and dispersion and precision measures (e.g., mean, median, SD, SEM, confidence intervals; and, for the major substantive results, a measure of effect size (e.g., Pearson's r, Cohen's d)
- Report exact p-values wherever possible alongside the summary statistics and 95% confidence intervals. These should be reported for all key questions and not only when the p-value is less than 0.05.

Please outline where this information can be found within the submission (e.g., sections or figure legends), or explain why this information doesn’t apply to your submission:

Each figure reports the statistical analysis in its legend, and the statistical analyses are explained in detail in the main text and methods.

(For large datasets, or papers with a very large number of statistical tests, you may upload a single table file with tests, Ns, etc., with reference to sections in the manuscript.)

**Group allocation**

- Indicate how samples were allocated into experimental groups (in the case of clinical studies, please specify allocation to treatment method); if randomization was used, please also state if restricted randomization was applied
- Indicate if masking was used during group allocation, data collection and/or data analysis

Please outline where this information can be found within the submission (e.g., sections or figure legends), or explain why this information doesn’t apply to your submission:

Trajectories were grouped based on the cell line from which they originated. For example, we analyzed trajectories from a U2OS RARA-HaloTag knock-in clonal cell line as a single pool.

**Additional data files (“source data”)**

- We encourage you to upload relevant additional data files, such as numerical data that are represented as a graph in a figure, or as a summary table
- Where provided, these should be in the most useful format, and they can be uploaded as “Source data” files linked to a main figure or table
- Include model definition files including the full list of parameters used
- Include code used for data analysis (e.g., R, MatLab)
- Avoid stating that data files are “available upon request”

Please indicate the figures or tables for which source data files have been provided:

- For Figure 5, we have provided the raw and labelled gel slices as Figure 5-Source Data 1.

- For Figure 6-Figure Supplement 1, we have provided the raw and labelled gel slices as Figure 6-Figure Supplement 1-Source Data 1.

- All trajectories from SPT experiments have been made available as a Dryad dataset (<https://doi.org/10.6078/D13H6N>). These are relevant to Figure 4, Figure 5, and their associated figure supplements.

- The code used to create trajectories from raw spaSPT movies by detecting and tracking fluorescent emitters has been made publicly available as a GitHub repository (<https://github.com/alecheckert/quot>)

- State arrays are publicly available as as a Python package (saspt) hosted on PyPI; the source code is available at <https://github.com/alecheckert/saspt>.

- The code used to generate non-optical trajectory simulations has been made publicly available as a GitHub repository (<https://github.com/alecheckert/strobesim>)

- The source code used to generate dynamic and optical simulations has been made publicly available as the sptPALMsim package (<https://github.com/alecheckert/sptpalmsim>).

- An implementation of the Dirichlet process mixture model (DPMM) used in this manuscript has been made publicly available at (<https://github.com/alecheckert/dpsp>).
